# Ginsenoside Rg3 attenuates myocardial ischemia/reperfusion-induced ferroptosis via the keap1/Nrf2/GPX4 signaling pathway

**DOI:** 10.1186/s12906-024-04492-4

**Published:** 2024-06-26

**Authors:** GuoFu Zhong, Junteng Chen, Yangtao Li, Yue Han, Maosheng Wang, Qinqi Nie, Mujuan Xu, Qinghua Zhu, Xiao Chang, Ling Wang

**Affiliations:** 1 The Fourth Clinical Medical College, Guangzhou University of Chinese Medicine, No. 1 Fuhua Road, Futian District, Shenzhen, 518000 China; 2 Department of intensive care unit, Shenzhen Traditional Chinese Medicine Hospital, Shenzhen, 518000 China

**Keywords:** Ginsenoside Rg3, Myocardial ischemia/reperfusion, Ferroptosis, The keap1/Nrf2/GPX4 signaling pathway

## Abstract

**Background:**

Ginsenoside Rg3 is a component of ginseng that protects against myocardial ischemia/reperfusion (MI/R) injury. Ferroptosis is a new form of cell death characterized by oxidative damage to phospholipids. The purpose of this study was to examine the role and of ginsenoside Rg3 in MI/R and the mechanism.

**Methods:**

A mouse model of left anterior descending (LAD) ligation-induced myocardial ischemia/reperfusion (MI/R) injury and oxygen-glucose deprivation/reperfusion (OGD/R) were used as in vitro and in vivo models, respectively. Echocardiographic analysis, 2,3,5-triphenyltetrazolium chloride (TTC) staining and hematoxylin-eosin (H&E) staining were used to assess the cardioprotective effects of ginsenoside Rg3. Western blotting, biochemical analysis, small interfering RNA analysis and molecular docking were performed to examine the underlying mechanism.

**Results:**

Ginsenoside Rg3 improved cardiac function and infarct size in mice with MI/R injury. Moreover, ginsenoside Rg3 increased the expression of the ferroptosis-related protein GPX4 and inhibited iron deposition in mice with MI/R injury. Ginsenoside Rg3 also activated the Nrf2 signaling pathway. Ginsenoside Rg3 attenuated myocardial ischemia/reperfusion-induced ferroptosis via the Nrf2 signaling pathway. Notably, ginsenoside Rg3 regulated the keap1/Nrf2 signaling pathway to attenuate OGD/R-induced ferroptosis in H9C2 cells. Taken together, ginsenoside Rg3 attenuated myocardial ischemia/reperfusion-induced ferroptosis via the keap1/Nrf2/GPX4 signaling pathway.

**Conclusions:**

Our findings demonstrated that ginsenoside Rg3 ameliorate MI/R-induced ferroptosis via the keap1/Nrf2/GPX4 signaling pathway.

**Supplementary Information:**

The online version contains supplementary material available at 10.1186/s12906-024-04492-4.

## Background

Myocardial ischemia is the root cause of heart failure due to myocardial infarction and inflammatory changes, which often lead to irreversible heart damage [1]. Timely and effective myocardial reperfusion, such as via percutaneous coronary interventional therapy (PPCI) or thrombolytic therapy, is an effective strategy for alleviating myocardial ischemic injury [2, 3]. However, myocardial ischemia/reperfusion (MI/R) can damage the heart muscle, which can contribute to further cardiomyocyte death.

Ferroptosis is a novel iron-dependent form of cell death characterized by oxidative damage to phospholipids and is related to iron ion metabolism, lipid oxidative metabolism and glutathione metabolic pathways [4, 5]. Previous studies have shown that oxidative stress and metabolic disorders are associated with the progression of MI/R [6]. In addition, increased levels of reactive oxygen species (ROS) have been observed in MI/R, and oxidative stress triggered by excessive ROS accumulation is an essential initiator of ferroptosis [7]. Under physiological conditions, cellular iron uptake is mainly controlled by transferrin receptor (TFR), which imports iron bound to transferrin from extracellular to intracellular via receptor-mediated endocytosis [8]. Whereas excess iron is stored in ferritin, an iron storage protein complex that includes ferritin light chain (FTL) and ferritin Heavy Chain 1 (FTH1) [9]. FTH1/FTL can increase iron levels through autophagic degradation, whereas a decrease in ferritin expression, especially its heavy chain, facilitates myocardial ferroptosis in myocardial ischemia/reperfusion injury [10]. Notably, glutathione peroxidase 4 (GPX4), which plays a central enzymatic role in ferroptosis, catalyzes the conversion of lipid hydroperoxides to nontoxic lipohydrols to prevent ferroptosis [4]. In myocardial ischemia reperfusion injury, increasing of GPX4 expression inhibits the accumulation of lipid peroxides, which in turn inhibits cardiomyocyte ferroptosis, thereby ameliorating MI/R injury [11].

Nuclear factor erythroid 2-related factor 2 (Nrf2) is a transcription factor that protects the myocardium from oxidative damage by activating antioxidant proteins [12]. Kelch-like ECH-associated protein 1 (keap1) is a chaperone protein that plays an essential role in maintaining redox homeostasis in vivo [13]. Under physiological conditions, Nrf2 binds to Keap1 in the cytoplasm and is continuously degraded by ubiquitination, as a result, it is unable to enter the nucleus to mediate transcriptional activity [14]. When oxidative stress occurs, conformational changes in keap1 result in the inability of Nrf2 to be degraded by ubiquitination [15]. Subsequently, Nrf2 translocates to the nucleus to activate various antioxidant genes, such as GPX4, HO-1 and NQO1, by binding to antioxidant response elements, ultimately reducing ROS production in vivo [16, 17]. A reduction in ROS ameliorates oxidative damage, which reduces lipid peroxidation and ferroptosis [18]. Thus, activating Nrf2 to reduce lipid peroxidation and ferroptosis may be an effective strategy to ameliorate MI/R.

Ginsenoside Rg3, which is the active ingredient in ginseng, has a variety of pharmacological effects, including anti-inflammatory and antioxidant effects [19, 20]. Recent pharmacological studies have shown that ginsenoside Rg3 can inhibit myocardial infarct size, maintain left ventricular function and ameliorate myocardial ischemia‒reperfusion injury [21]. In 2021, it was reported that ginsenoside Rg3 improved transverse aortic constriction (TAC)-induced myocardial hypertrophy in rats [22] and inhibited coronary artery ligation (CAL)-induced heart failure in mice [23]. In 2023, ginsenoside Rg3 was reported to ameliorate myocardial fibrosis after infarction induced by left anterior descending coronary artery ligation in mice [24]. However, it is not clear whether ginsenoside Rg3 ameliorates myocardial ischemia‒reperfusion injury by inhibiting ferroptosis. Therefore, we investigated the pharmacological effects of ginsenoside Rg3 on MI/R and the potential mechanisms.

## Materials and methods

### Reagents

Ginsenoside Rg3 (purity > 98%, B21059) was obtained from Shenzhen Combe Scientific Instrument Co., Ltd. (Shenzhen, China). Antibodies against keap1 (AF5266), GPX4 (DF6701), HO-1 (AF5393) and β-tubulin (AF7011) were purchased from Affinity Biosciences (Jiangsu, China). FTH1 (#3998S), Lamin B (#13435S) and Nrf2 (#20733S) antibodies were obtained from Cell Signaling Technology (Boston, MA). β-actin (AF2811) was purchased from Beyotime (Shanghai, China). NQO1 (PAB32354) was purchased from Bioswamp (Wuhan, China). HRP-linked anti-mouse IgG (S0002) and HRP-linked anti-rabbit IgG (S0001) were purchased from Affinity Biosciences (Jiangsu, China).

### Animals and the MI/R model

The experimental procedures were approved by the Animal Ethical Use Committee of Guangzhou University of Chinese Medicine (No. 00371405). Male C57BL/6 mice (6–8 weeks) were purchased from the Laboratory Animal Center of Guangzhou University of Chinese Medicine. The mice were subjected to MI/R as previously described. Briefly, the mice were anesthetized by i.p. injection of 0.3% sodium pentobarbital and ventilated with a pressure-control ventilator. The left anterior descending coronary artery was ligated with a 7 − 0 silk suture and a PE10 tube for 30 min. After 30 min of ischemia, the suture and the PE10 tube were removed. At the end of 6 h of reperfusion. The mice were pretreated with ginsenoside Rg3 and normal saline for 7 days before ischemia. The mice were randomly divided into five groups: the sham group was given saline, the model group was given saline, and the MI/R + Ginsenoside Rg3 groups were given 5, 10 or 20 mg/kg ginsenoside Rg3. At the end of the experiment, the mice were euthanized by cervical dislocation, which resulted in immediate unconsciousness and cessation of breathing.

### 2,3,5-Triphenyltetrazolium chloride (TTC) staining

The hearts were removed, frozen, sliced and incubated with 1% TTC at 37 °C for 15 min. The area of myocardial infarction in each section was analyzed by imaging software.

### Echocardiographic analysis

The mice were anesthetized with isoflurane ventilation, and an echocardiography system (Visual Sonics Inc., Toronto, Canada) was used to assess cardiac function after MI/R. The left ventricular ejection fraction (LVEF) and left ventricular shortening (LVFS) were calculated.

### Hematoxylin-eosin (H&E) staining

The hearts were fixed with 4% paraformaldehyde, embedded in paraffin, cut into 5 μm slices, stained with hematoxylin-eosin (H&E) and observed under a light microscope.

### Cell culture and oxygen-glucose deprivation/reperfusion (OGD/R) model

H9C2 cardiomyocytes were obtained from BeiNa Biological Technology Co., Ltd. and cultured in DMEM supplemented with 10% FBS (Invitrogen) at 37 °C in a 5% CO_2_ incubator. H9C2 cells were subjected to hypoxic conditions (0.1% O_2_, 5% CO_2_ and 95% N_2_) for 6 h at 37 °C. The cells were then reoxygenated for 24 h at 37 °C under normal culture conditions (95% air and 5% CO_2_). H9C2 cells were treated with the indicated doses of ginsenoside Rg3 (5, 10, 20 µM) and ML334 (50 µM) for 24 h. The cells were treated with erastin (10 µM) to induce ferroptosis.

### Biochemical analysis

Iron concentrations were determined with an iron assay kit (Elabscience) according to the manufacturer’s instructions.

Glutathione (GSH) levels were determined by a GSH assay kit (Nanjing Jiancheng Bioengineering Research Institute) according to the manufacturer’s instructions.

### Western blot analysis

H9c2 cells and the border zones of the infarcted hearts were lysed with RIPA lysis buffer (Beyotime, China) supplemented with protease and phosphatase inhibitors. The samples were separated by 10% or 12% sodium dodecyl sulfate‒polyacrylamide gel electrophoresis (SDS‒PAGE) and subsequently transferred to polyvinylidene difluoride (PVDF) membranes, which were blocked with TBST buffer containing 5% BSA for 2 h at room temperature and incubated with primary antibodies against β-tubulin, Lamin B, Nrf2, HO-1, FTH1, GPX4, NQO1, β-actin and Keap1 overnight at 4 °C, followed by incubation with the appropriate HRP-conjugated secondary antibodies diluted in 2% BSA in TBST. The bands were detected using ECL reagent. The grayscale of the protein bands was determined by ImageJ software.

### siRNA transfection

H9C2 cells were cultured in 6-well plates. Control siRNA and Nrf2 siRNA were transfected into the cells (Thermo Fisher Scientific, Waltham, MA, USA) according to the manufacturer’s instructions. After 24 h of culture, the cells were subjected to OGD/R, treated with ginsenoside Rg3 and then subjected to Western blot analysis.

### Molecular docking

The molecular structure of ginsenoside Rg3 was retrieved from PubChem Compound. The 3D coordinates of Keap1 (PDB ID, 7C60; resolution, 1.95 Å) were downloaded from the PDB database. For docking analysis, all protein and molecular files were converted into the PDBQT format; all water molecules were excluded, and polar hydrogen atoms were added. The grid box was centered to cover the domain of each protein and accommodate free molecular movement. The grid length was set to 30 Å × 30 Å × 30 Å, and the grid point distance was 0.05 nm. Molecular docking studies were performed by Autodock Vina 1.2.2 (http://autodock.scripps.edu/).

### Statistical analysis

Quantitative data are shown as the means ± SDs. Comparisons of among groups were performed using one-way analysis of variance with Dunnett’s test. For two independent variables, two-way ANOVA was used. The statistical data were analyzed using SPSS 21.0. P values < 0.05 indicated statistical significance.

## Results

### Ginsenoside Rg3 attenuated myocardial ischemia/reperfusion injury-induced damage to cardiac function

**Fig. 1:**
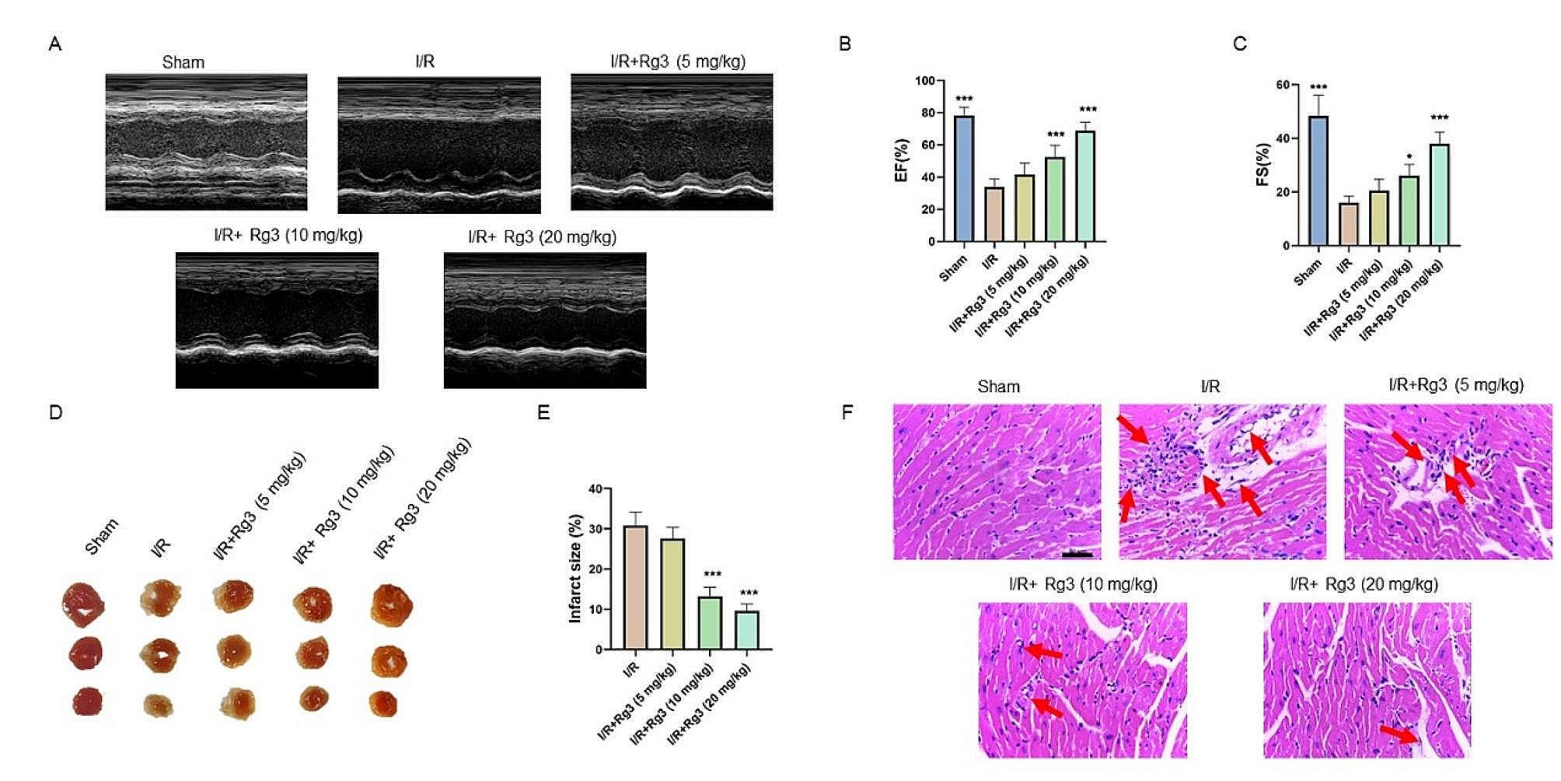
Ginsenoside Rg3 improved cardiac function and protected against myocardial ischemia/reperfusion (MI/R) injury in mice. (**A**) M-mode images of each group. (**B**) Ejection fraction (EF%). (**C**) Fractional shortening (FS%). (**D**) TTC results in each group of hearts. (**E**) Statistical analysis of the TTC results. (**F**) H&E-stained heart sections in each group. The data are shown as the means ± SDs (*n* = 5). Statistical comparisons between the different groups were performed by one-way ANOVA followed by Dunnett’s test. *p < 0.05, **p < 0.01, ***p < 0.001, the sham group or the I/R + Rg3 group vs. the I/R group

To verify the protective effect of ginsenoside Rg3 on cardiac tissues, we induced myocardial ischemia/reperfusion injury in mice and administered ginsenoside Rg3 by gavage. Cardiac function was impaired by MI/R injury, as evidenced by a decrease in the left ventricular ejection reduction fraction (LVEF) and left ventricular shortening reduction (LVFS). However, 10 and 20 mg/kg ginsenoside Rg3 ameliorated myocardial ischemia/reperfusion injury (Fig. 1A-C). As shown in Fig. 1D-E, ginsenoside Rg3 significantly reduced the MI/R-induced myocardial infarction area. H&E staining revealed that MI/R disrupted myofiber structure and led to inflammatory cell infiltration. However, ginsenoside Rg3 not only prevented changes in myofiber structure but also reduced the recruitment of inflammatory cells (Fig. 1F). Overall, ginsenoside Rg3 exerted cardioprotective effects.

### Ginsenoside Rg3 ameliorated ferroptosis induced by myocardial ischemia/reperfusion injury

**Fig. 2:**
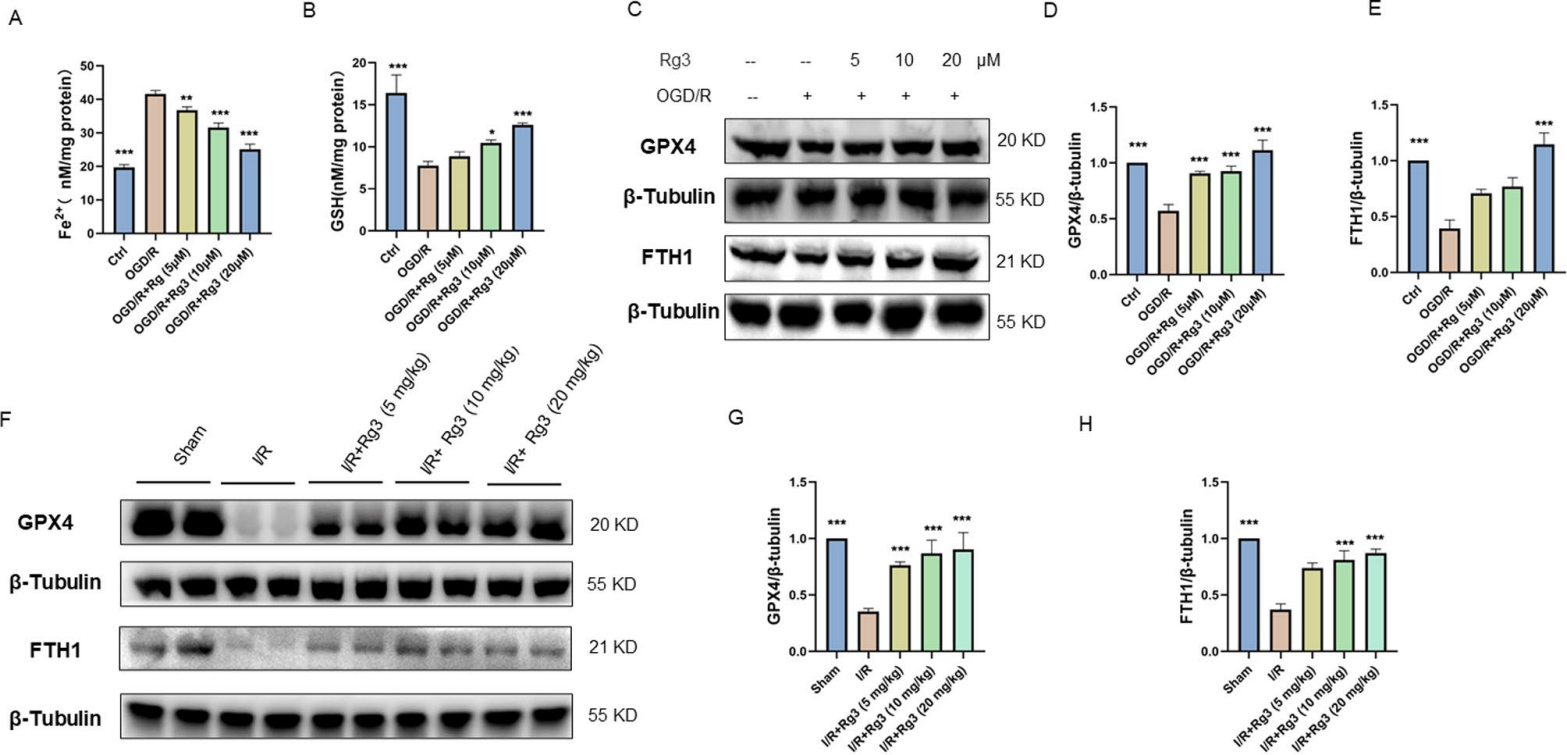
Effect of ginsenoside Rg3 on ferroptosis. (**A**, **B**) Determination of ferrous iron (Fe2+) and glutathione (GSH) levels in H9C2 cells. (**C**–**E**) The protein levels of GPX4 and FTH1 in H9C2 cells. The data are shown as the means ± SDs (*n* = 3). *p < 0.05, **p < 0.01, ***p < 0.001, the Ctrl group or OGD/R + Rg3 groups vs. the OGD/R group. (**F**-**H**) The protein levels of GPX4 and FTH1 in the heart tissue of the mice. The data are shown as the means ± SDs (*n* = 5). Statistical comparisons between the different groups were performed by one-way ANOVA followed by Dunnett’s test. *p < 0.05, **p < 0.01, ***p < 0.001, the sham group or the I/R + Rg3 group vs. the I/R group

Myocardial ischemia/reperfusion injury is accompanied by ferroptosis, which is characterized by iron accumulation, lipid peroxidation, and glutathione depletion [25]. In addition, studies have reported that ginsenoside Rg3 ameliorates acute pancreatitis by inhibiting ferroptosis [26]. The levels of iron and GSH in H9C2 cells were examined. As shown in Fig. 2A-B, ginsenoside Rg3 significantly reduced iron levels and increased GSH levels in H9C2 cells exposed to OGD/R conditions. In addition, GPX4 catalyzes the conversion of lipid peroxides to the lipohydrol form, which plays a central role in ferroptosis [4]. Ferritin heavy chain 1 (FTH1) is an iron storage protein that is essential for iron metabolism [9]. Accordingly, we examined the expression levels of ferroptosis-related proteins. The Western blot results showed that ginsenoside Rg3 promoted the expression of GPX4 and FTH1 in H9C2 cells exposed to OGD/R conditions (Fig. 2C-E). The changes in GPX4 and FTH1 protein expression in mice with myocardial ischemia/reperfusion injury was consistent with the results in cells (Fig. 2F-H). These results indicated that ginsenoside Rg3 inhibited MI/R-induced ferroptosis.

### The ferroptosis inducer erastin reversed the protective effect of ginsenoside Rg3 against OGD/R-induced injury in H9C2 cardiomyocytes

**Fig. 3:**
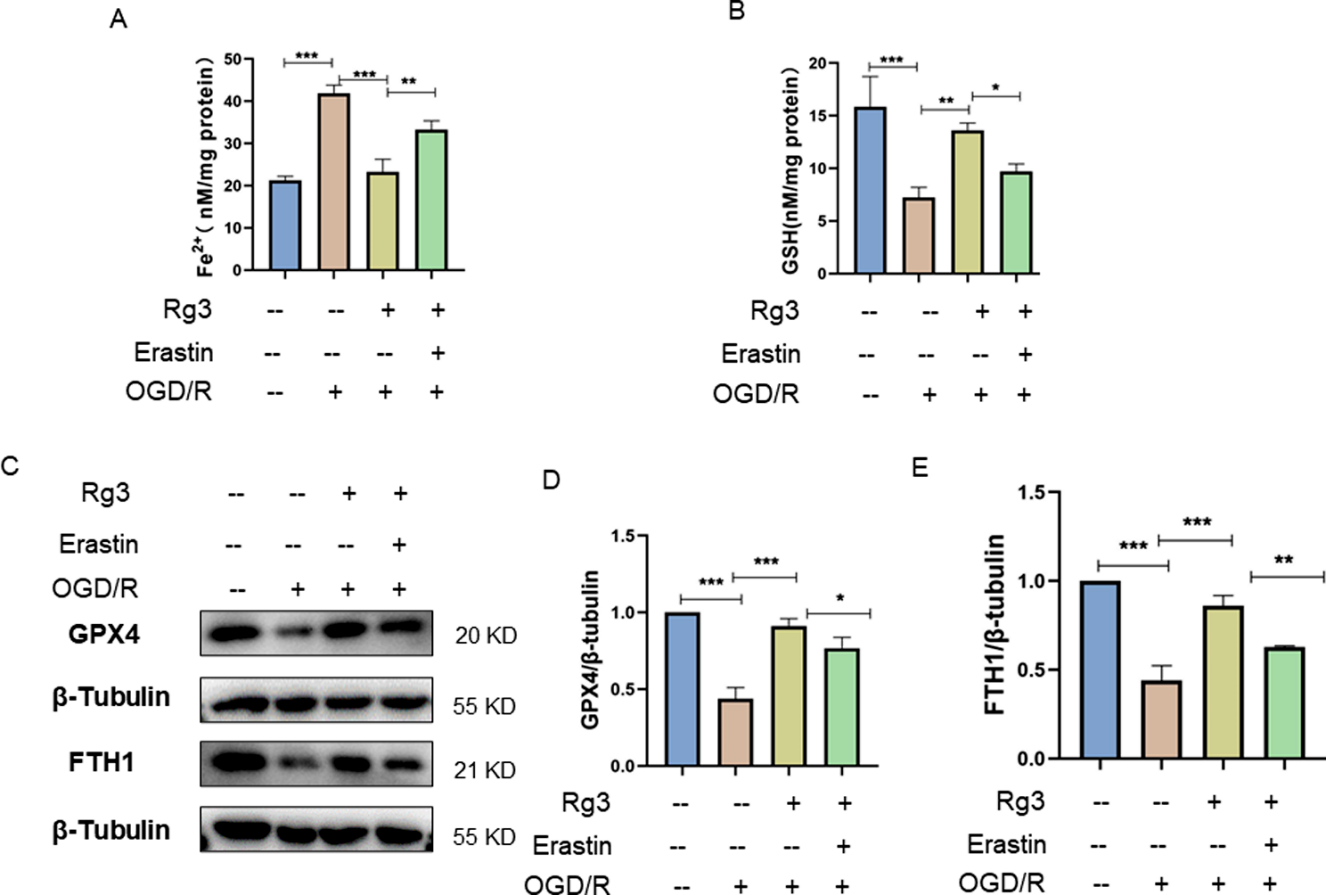
Erastin reversed the protective effect of ginsenoside Rg3 against OGD/R-induced injury in H9C2 cardiomyocytes. (**A**, **B**) Ferrous iron (Fe2+) and glutathione (GSH) levels in H9C2 cells were examined by kits. (**C**–**E**) The protein levels of GPX4 and FTH1 in H9C2 cells were examined by Western blotting. The data are shown as the means ± SDs (*n* = 3). *p < 0.05, **p < 0.01, ***p < 0.001, the Ctrl group, the OGD/R + Rg3 group or the OGD/R + Rg3 + Erastin group vs. the OGD/R group

To further validate the role of ferroptosis in myocardial ischemia/reperfusion injury, we used erastin to induce ferroptosis in H9C2 cells exposed to OGD/R conditions. As shown in Fig. 3A-B, ginsenoside Rg3 decreased the iron content and increased the GSH level in H9C2 cells exposed to OGD/R. However, erastin partially reversed these changes. Western blot analysis indicated that ginsenoside Rg3 promoted the expression of GPX4 and FTH1 in H9C2 cells exposed to OGD/R conditions. However, the promotion of ferroptosis-related protein expression by ginsenoside Rg3 was partially inhibited by erastin (Fig. 3C-E). These results showed that erastin reversed the inhibitory effect of ginsenoside Rg3 on OGD/R-induced ferroptosis in H9C2 cells.

### Ginsenoside Rg3 regulated the Nrf2 signaling pathway to protect against OGD/R-induced injury in H9C2 cardiomyocytes

**Fig. 4:**
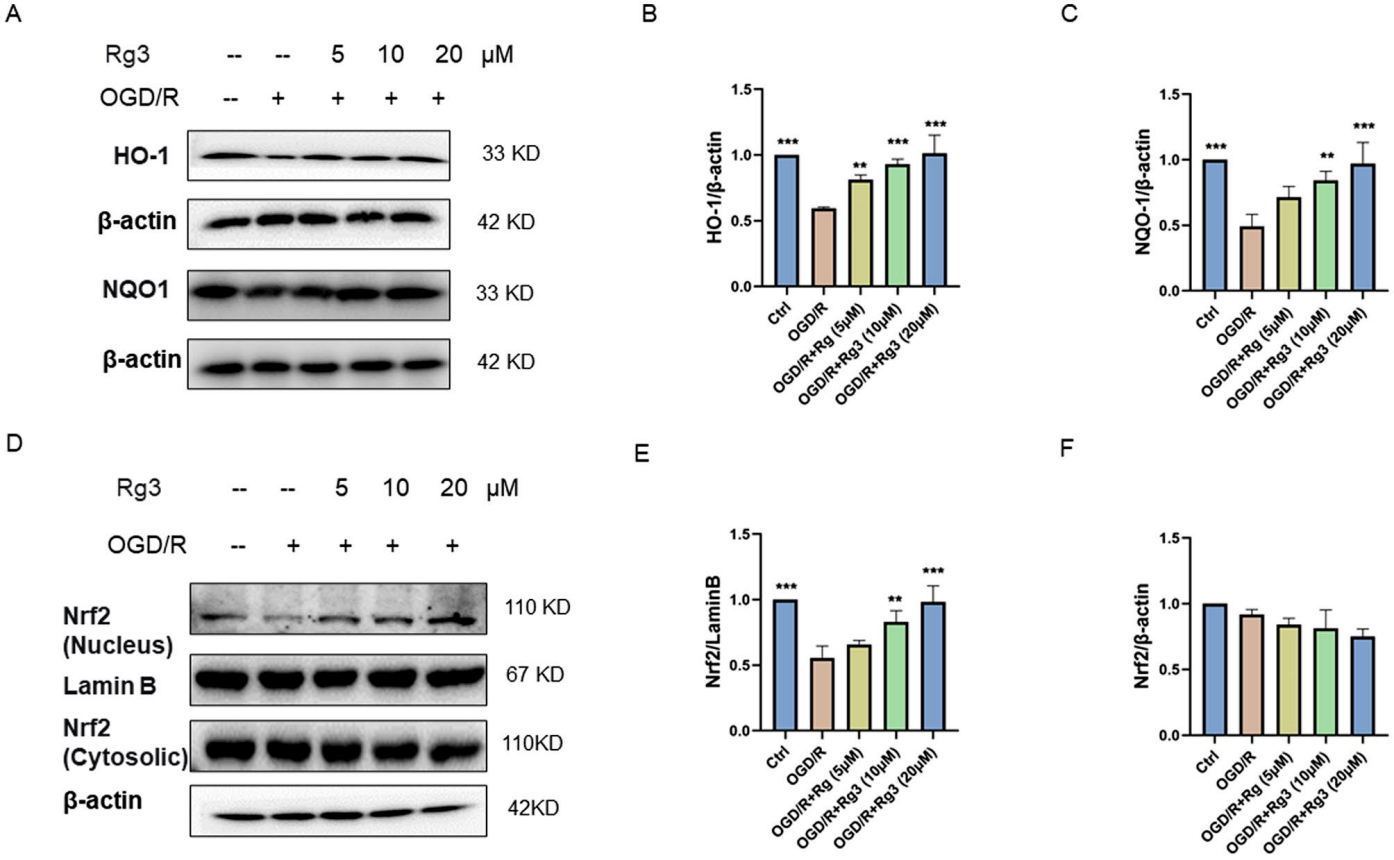
Ginsenoside Rg3 regulates the Nrf2 signaling pathway. (**A**-**C**) The protein levels of HO-1 and NQO1 in H9C2 cells were examined by Western blotting. (**D**) The proteins were isolated from the cytoplasm and nucleus for Western blot analysis. (**E**, **F**) Histogram of Nrf2 protein expression in the nucleus and cytoplasm. The data are shown as the means ± SDs (*n* = 3). *p < 0.05, **p < 0.01, ***p < 0.001, the Ctrl group or the OGD/R + Rg3 group vs. the OGD/R group

Myocardial ischemia/reperfusion injury is closely related to oxidative stress [27]. When oxidative stress occurs, Nrf2 translocates to the nucleus and activates various antioxidant genes, such as HO-1 and NQO1, ultimately exerting antioxidant effects [28]. Previously, ginsenoside Rg3 was reported to attenuate cerebral ischemia/reperfusion injury by mitigating mitochondrial oxidative stress via the Nrf2/HO-1 signaling pathway [29]. Therefore, we examined the expression of the antioxidant proteins HO-1 and NQO1. As shown in Fig. 4A-C, the expression of HO-1 and NQO1 was reduced in the OGD/R group; however, ginsenoside Rg3 increased the expression of these proteins. Moreover, we determined the effect of ginsenoside Rg3 on Nrf2 nuclear translocation. Western blot analysis revealed that ginsenoside Rg3 promoted Nrf2 translocation into the nucleus (Fig. 4D-F). Taken together, ginsenoside Rg3 modulated the Nrf2 signaling pathway during MI/R injury.

### Ginsenoside Rg3 attenuated OGD/R-induced ferroptosis in H9C2 cardiomyocytes via Nrf2

**Fig. 5:**
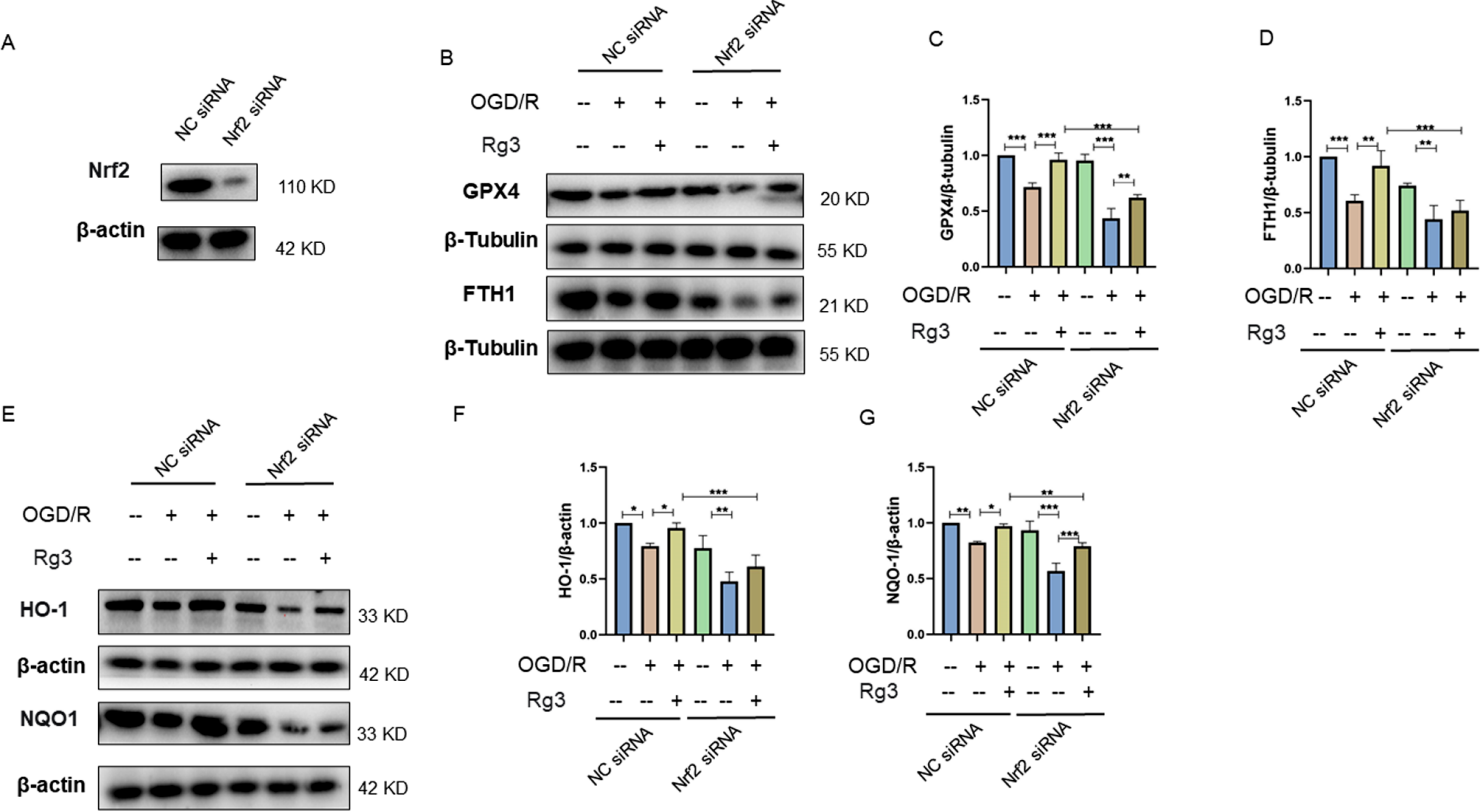
Ginsenoside Rg3 attenuates ferroptosis via Nrf2. (**A**) Western blot analysis of Nrf2 in H9C2 cells. (**B**-**D**) The protein levels of GPX4 and FTH1 in H9C2 cells after Nrf2 silencing were examined by Western blotting. (**E**-**G**) The protein levels of HO-1 and NQO1 in H9C2 cells after Nrf2 silencing were examined by Western blotting. The data are shown as the means ± SDs (*n* = 3). *p < 0.05, **p < 0.01, ***p < 0.001.) The OGD/R + NC siRNA group vs. the NC siRNA group or the OGD/R + Rg3 + NC siRNA group; the OGD/R + Nrf2 siRNA group vs. the Nrf2 siRNA group or the OGD/R + Rg3 + Nrf2 siRNA group; and the OGD/R + Rg3 + Nrf2 siRNA group versus the OGD/R + Rg3 + NC siRNA group

Nrf2 can alleviate ferroptosis by activating GPX4 [16]. Thus, we investigated whether the cardioprotective effect of ginsenoside Rg3 was related to the regulation of ferroptosis by the Nrf2 signaling pathway, and we used Nrf2-specific small interfering RNAs to knock down Nrf2 expression. As shown in Fig. 5A, Nrf2 was knocked down in H9c2 cells. In addition, ginsenoside Rg3-mediated promotion of the expression of ferroptosis-related proteins in H9C2 cells exposed to OGD/R conditions was partially blocked by Nrf2 knockdown (Fig. 5B-D). Moreover, we observed that knockdown of Nrf2 reduced the relative abundance of the antioxidant proteins HO-1 and NQO1 (Fig. 5E-G). These data demonstrated that Nrf2 was involved in the cardioprotective and anti-ferroptotic effects of ginsenoside Rg3.

### Ginsenoside Rg3 regulated the keap1/Nrf2 signaling pathway to attenuate OGD/R-induced ferroptosis in H9C2 cardiomyocytes

**Fig. 6:**
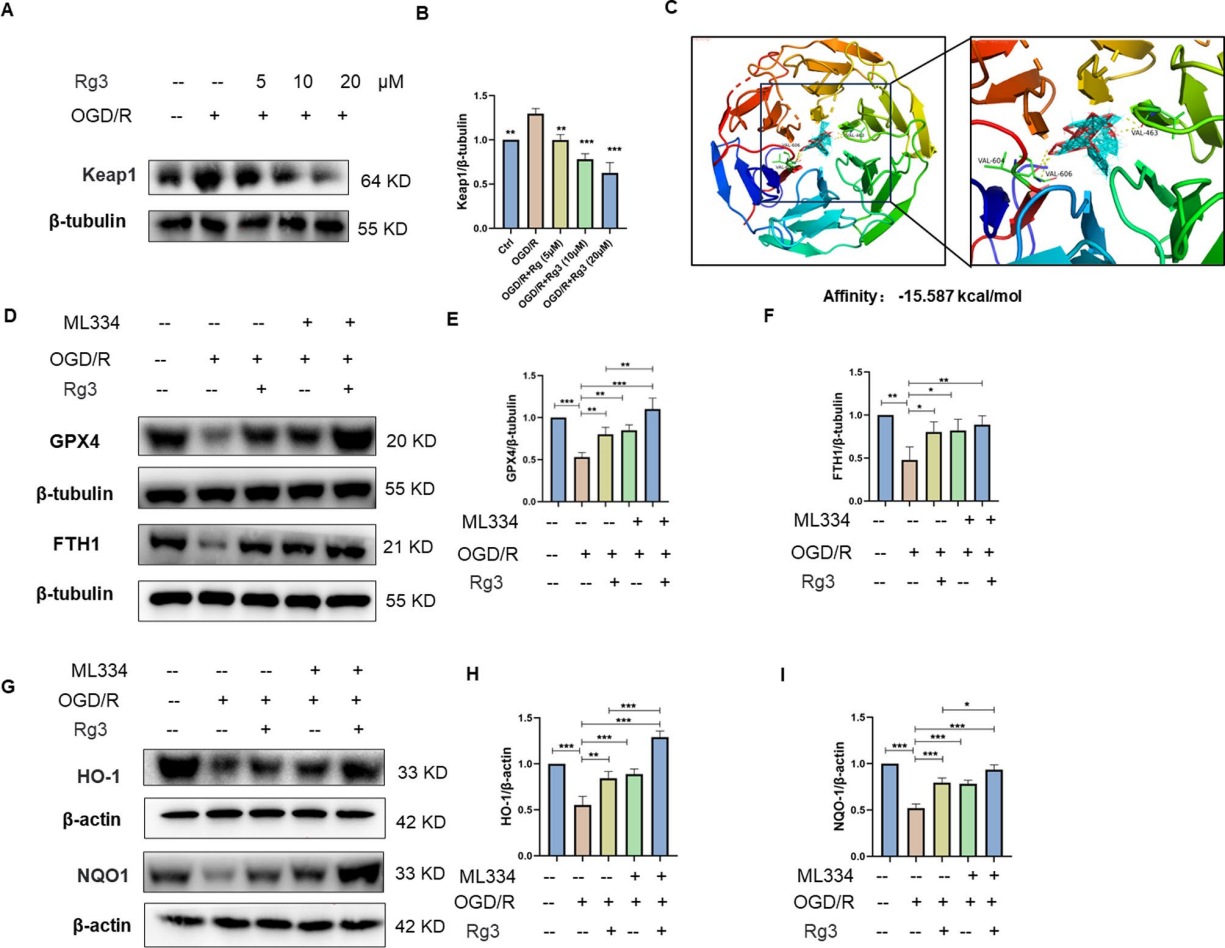
Ginsenoside Rg3 regulates the keap1/Nrf2 signaling pathway to attenuate OGD/R-induced ferroptosis. (**A**, **B**) The protein level of keap1 in H9C2 cells was examined by Western blotting. (**C**) Molecular docking analysis of ginsenoside Rg3 with the Kelch domain of Keap1. (**D**-**F**) The protein levels of GPX4 and FTH1 in H9C2 cells treated with ML334 were examined by Western blotting. (**G**-**I**) The protein levels of HO-1 and NQO1 in H9C2 cells treated with ML334 were examined by Western blotting. The data are shown as the means ± SDs (*n* = 3). *p < 0.05, **p < 0.01, ***p < 0.001. The Ctrl group, the OGD/R + Rg3 group, the OGD/R + ML334 group or the OGD/R + ML334 + Rg3 group vs. the OGD/R group. The OGD/R + Rg3 group or the OGD/R + ML334 group vs. the OGD/R + ML334 + Rg3 group

Under physiological conditions, Nrf2 binds to Keap1 in the cytoplasm resulting in the inability to enter the nucleus to exert transcriptional activity [30]. However, it is not clear whether ginsenoside Rg3 can target keap1, causing a conformational change in keap1 that leads to the translocation of Nrf2 to the nucleus. We first examined Keap1 expression in H9C2 cells exposed to OGD/R conditions. The results revealed that ginsenoside Rg3 inhibited keap1 expression (Fig. 6A, B). To evaluate the affinity of ginsenoside Rg3 for keap1, we performed molecular docking analysis. The results showed that ginsenoside Rg3 bound to Keap1 through hydrogen bonds and strong hydrophobic interactions and had a low binding energy of -15.587 kcal/mol, indicating highly stable binding (Fig. 6C). To further clarify whether ginsenoside Rg3 ameliorated ferroptosis induced by myocardial ischemia/reperfusion injury via the keap1/Nrf2 signaling pathway, we used ML334, a keap1/Nrf2 interaction inhibitor. As shown in Fig. 6D-F, treatment with ginsenoside Rg3 and ML334 more robustly promoted ferroptosis-related protein expression than treatment with ginsenoside Rg3 or ML334 alone. Similarly, treatment with ginsenoside Rg3 and ML334 promoted antioxidant-related protein expression (Fig. 6G-I). Overall, ginsenoside Rg3 regulates the keap1/Nrf2 signaling pathway to attenuate OGD/R-induced ferroptosis in H9C2 cells.

## Discussion

Myocardial ischemia/reperfusion injury (MI/R) poses a serious threat to human health, exhibits a high mortality rate in critical patients and imposes a heavy burden on the public [31], and it is imperative to develop treatment strategies and examine its pathogenesis. Ginsenoside Rg3 is a well-studied ginsenoside with high pharmacological activity that has anticancer and antiangiogenic effects [32, 33], but its role in MI/R has rarely been reported. We showed that ginsenoside Rg3 significantly improved cardiac function and infarct size in mice with myocardial ischemia/reperfusion injury.

Ferroptosis is an iron-dependent form of cell death that is implicated in myocardial ischemia/reperfusion injury [4]. Ferroptosis is associated with the metabolism of iron, glutathione, and ROS [34]. Extracellular Fe^3+^ is transported into the cell by transferrin, reduced to Fe^2+^, and reacts with excess H_2_O_2_ in the cell to produce large amounts of ROS, thereby triggering ferroptosis [35]. Multiple studies have shown that ROS production is increased during ischemia/reperfusion injury [36]. During the ischemic phase, ROS accumulation in cells decreases the effect of antioxidants [37]. After the blood supply to ischemic tissues is restored, oxidative stress contributes to cell damage and death, depending on the severity of ROS levels [7]. In contrast, cellular resistance to ROS is mediated by a variety of antioxidant molecules and enzymes, including the glutathione (GSH)-dependent antioxidant system [1, 38]. Glutathione (GSH), which is an antioxidant, scavenges oxygen free radicals in the body, which reduces the damage caused by oxidative stress [39]. Consequently, the upregulation of GSH during myocardial ischemia/reperfusion injury may attenuate the damage caused by oxygen free radicals to cardiomyocytes and reduce the extent of reperfusion injury [7]. Notably, our results revealed that ginsenoside Rg3 promoted GSH expression. GPX4 plays a critical regulatory role in ferroptosis by converting glutathione to oxidized glutathione and reducing lipid peroxides to nontoxic alcohols [5]. It has been shown that inhibiting GPX4 activity causes the accumulation of lipid peroxides, leading to ferroptosis [16]. In addition, studies have reported that ginsenoside Rg3 ameliorates acute pancreatitis by inhibiting ferroptosis [26]. In contrast, Hu et al. reported that ginsenoside Rg3 promoted hepatic stellate cell ferroptosis to suppress liver fibrosis progression [40]. However, how ginsenoside Rg3 affects ferroptosis to ameliorate myocardial ischemia‒reperfusion injury is unknown. Accordingly, we examined the effect of ginsenoside Rg3 on cardiomyocyte ferroptosis. We found that Ginsenoside Rg3 ginsenoside inhibited iron deposition and promoted the expression of the ferroptosis-associated proteins GPX4 and FTH1, thereby exerting a protective effect on cardiomyocytes. Furthermore, the ferroptosis inducer erastin partially reversed the protective effect of ginsenoside Rg3 on OGD/R-induced H9C2 cardiomyocytes.

The keap1/Nrf2 signaling pathway is a major defense mechanism against oxidative and electrophilic stress [18]. Keap1 modulates the ubiquitination and translocation of Nrf2 into the nucleus [41, 42]. Previous studies have shown that the structures of chemicals that induce oxidative stress have an electrophilic center that allows them to react with cysteine residues in proteins [18, 43]. In the current study, molecular docking suggested that ginsenosides Rg3 and Keap1 have high affinity. Nrf2, which is a transcriptional regulator, activates adaptive responses like anti-oxidative stress and ferroptosis by transcriptionally inducing a multitude of antioxidant enzymes such as GPX4 and HO-1 [44]. In addition, activation of the Nrf2 signaling pathway is a major mechanism of the cellular defense against OGD/R induction [45]. Previously, ginsenoside Rg3 was reported to attenuate cerebral ischemia‒reperfusion injury by mitigating mitochondrial oxidative stress via the Nrf2/HO-1 signaling pathway [29]. However, whether ginsenoside Rg3 alleviates myocardial ischemia/reperfusion injury via the Nrf2 signaling pathway is not clear. Our results indicated that ginsenoside Rg3 activated the Nrf2 signaling pathway to protect against MI/R- and OGD/R-induced oxidative stress and ferroptosis. Silencing Nrf2 significantly partially blocked the inhibitory effect of ginsenoside Rg3 on OGD/R-induced ferroptosis in cardiomyocytes. Interestingly, when ML334 was used to inhibit the interaction between keap1 and Nrf2, ginsenoside Rg3 robustly promoted the expression of ferroptosis- and antioxidative proteins. Ginsenoside Rg3 regulates the keap1/Nrf2 signaling pathway to inhibit ferroptosis. In this study, we confirmed the protective effect of ginsenoside Rg3 on H9C2 cardiomyocytes. We will further verify this finding in other cardiac cells in addition to H9C2 cells at a later stage. In addition, ginsenoside Rg3 inhibits ferroptosis induced by other pathways during myocardial ischemia‒reperfusion injury, which we will also further validate. As mentioned previously, ginsenoside Rg3 protect the heart from ischemia/reperfusion injury via the Keap1/Nrf2/GPX4 pathway both in vivo and in vitro.

## Conclusions

**Fig. 7:**
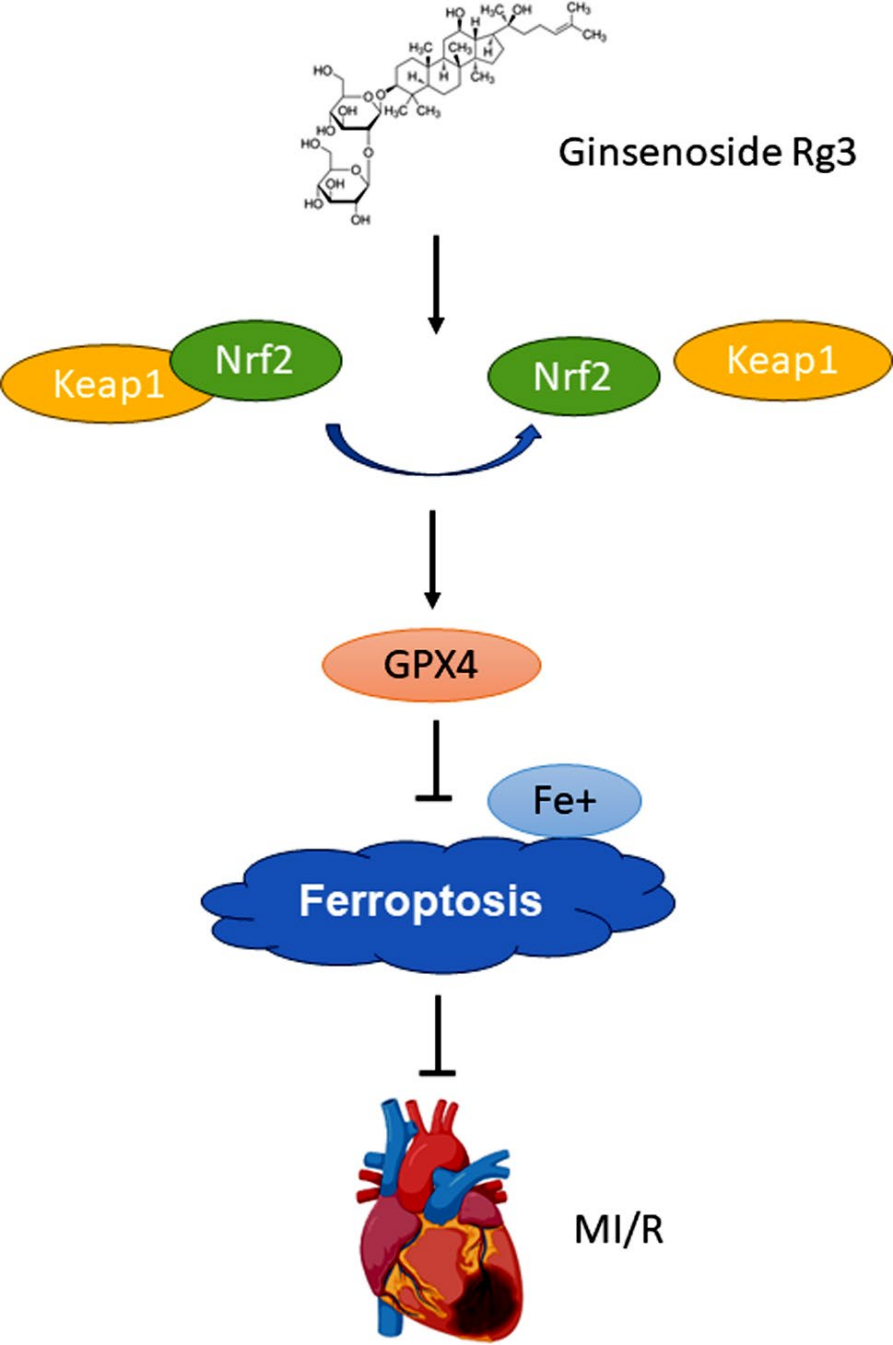
Ginsenoside Rg3 attenuates myocardial ischemia/reperfusion-induced ferroptosis via the keap1/Nrf2/GPX4 signaling pathway

In conclusion, we demonstrated that ginsenoside Rg3 ameliorates myocardial ischemia/reperfusion injury in mice by suppressing ferroptosis. Furthermore, our results demonstrated that ginsenoside Rg3 attenuates myocardial ischemia/reperfusion-induced ferroptosis via the keap1/Nrf2/GPX4 signaling pathway (Fig. 7).

### Electronic supplementary material

Below is the link to the electronic supplementary material.

Supplementary Material 1

## Data Availability

All relevant study data are included in the article and the supplementary materials.

## Abbreviations

FTH1: Ferritin heavy chain 1
GPX4: Glutathione peroxidase 4
GSH: Glutathione
H&E: Hematoxylin-eosin
Keap1: Kelch-like ECH-associated protein 1
LAD: Left anterior descending
LVEF: Left ventricular ejection fraction
LVFS: Left ventricular shortening
MI/R: Myocardial ischemia/reperfusion
Nrf2: Nuclear factor erythroid 2-related factor 2
OGD/R: Oxygen-glucose deprivation/reperfusion
PPCI: Percutaneous coronary interventional therapy
PVDF: Polyvinylidene difluoride
SDS-PAGE: Sodium dodecyl sulfate polyacrylamide gel electrophoresis
TTC: 2,3,5-triphenyltetrazolium chloride
