# Supplementary material for: Ginsenoside Rg3 attenuates myocardial ischemia/reperfusion-induced ferroptosis via the keap1/Nrf2/GPX4 signaling pathway
Source: BMC Complement Med Ther. 2024 Jun 26;24:247. doi: 10.1186/s12906-024-04492-4 (PMC11209975; doi:10.1186/s12906-024-04492-4)
Supplement: Supplementary file 1 — Supplementary Material 1 [file 12906_2024_4492_MOESM1_ESM.docx]

**Ginsenoside Rg3 attenuates** **myocardial ischemia/reperfusion-induced ferroptosis via the keap1/Nrf2/GPX4 signaling pathway**

**GuoFu Zhong^1,2^, Junteng Chen^1,2^, Yangtao Li^1,2^, Yue Han^1,2^, Maosheng Wang^1,2^, Qinqi Nie^1,2^, Mujuan Xu^1,2^, Qinghua Zhu^1,2^, Xiao Chang^1,2*^,** **Ling Wang^1,2*^**

**Response:** We cut the blots before hybridizing with the antibody, internal reference blot and the destination blot are from the same membrane. In addition, we have labeled each lane with the name of the sample in that lane, the standard protein size markers with the expected molecular weight and ensured it is clear what proteins are presented on each gel.

**Figure2**

**Rg3 -- -- 5 10 20 μM**

**OGD/R -- + + + +**


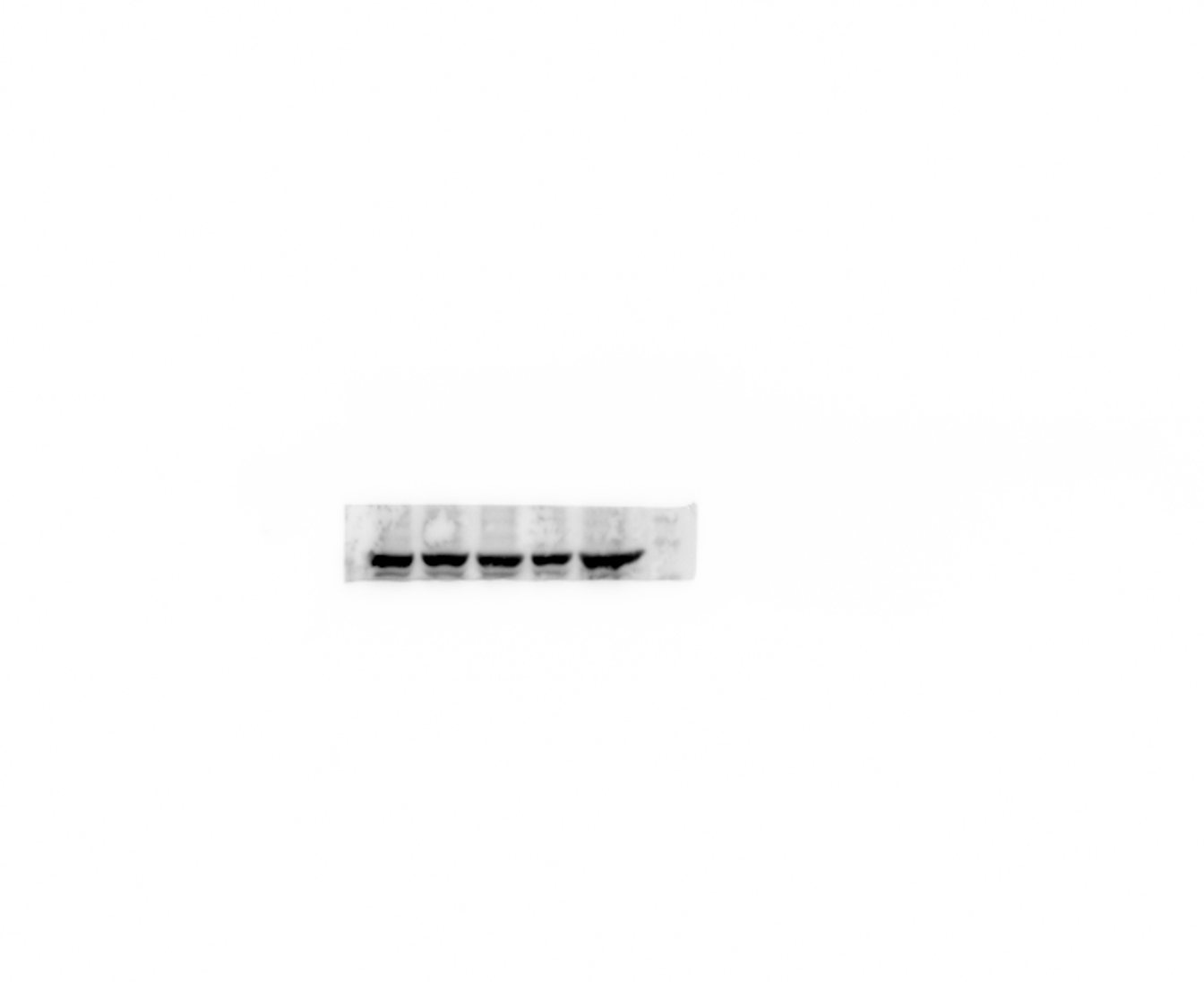


**GPX4**

20 KD

**β-Tubulin**





55 KD





21 KD

**FTH1**





**β-Tubulin**

55 KD

**β-Tubulin**

**Raw data for figure2C in the manuscript.**

**Raw data for figure2F in the manuscript.**

I/R+ Rg3 (10 mg/kg)

I/R+Rg3 (5 mg/kg)

I/R+ Rg3 (20 mg/kg)

I/R

Sham





**GPX4**

20 KD

**β-Tubulin**





55 KD

I/R+ Rg3 (20 mg/kg)

I/R+ Rg3 (10 mg/kg)

I/R+Rg3 (5 mg/kg)

Sham

I/R

**Raw data for figure2F**

**in the manuscript.**

**FTH1**





21 KD

**β-Tubulin**





55 KD

**Figure3**

**Raw data for figure3C in the manuscript.**

**β-Tubulin**

**β-Tubulin**

**GPX4**

**Rg3 -- -- + +**

**Erastin -- -- -- +**

**OGD/R -- + + +**





20 KD





55KD





**FTH1**

21 KD





55 KD

**Figure4**

**β-actin**

**Rg3 -- -- 5 10 20 μM**





**OGD/R -- + + + +**

33 KD

**HO-1**





42 KD





**NQO1**

33 KD





**β-actin**

42 KD





**Nrf2(Nucleus)**

110 KD





67 KD

**Lamin B**





**Nrf2(Cytosolic)**

110 KD





42 KD

**β-actin**

**Raw data for figure4A and 4D in the manuscript.**

**Figure5**



NC siRNA

Nrf2 siRNA

**Nrf2**

**Raw data for figure5A in the manuscript.**

110 KD


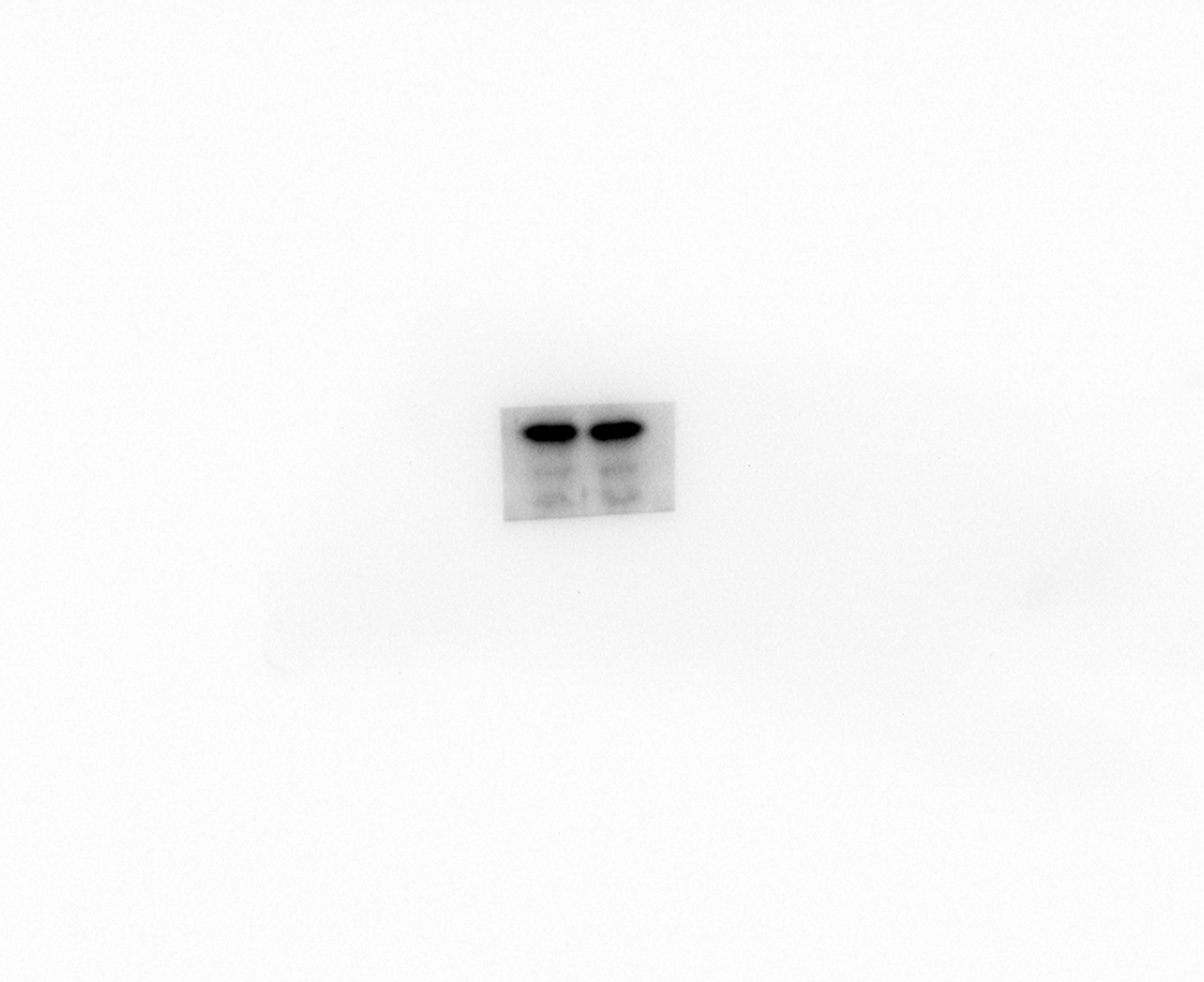


**β-actin**

42 KD

NC siRNA

Nrf2 siRNA

**OGD/R -- + + -- + +**

**Rg3 -- -- + -- -- +**

**HO-1**

**β-actin**

**β-actin**

**FTH1**

**GPX4**





20 KD





**β-Tubulin**

55 KD





21 KD





**β-Tubulin**

55 KD





33 KD





42 KD

42 KD

**NQO1**





33 KD





**Raw data for figure5B and 5E in the manuscript.**

**Raw data for figure6A in the manuscript.**

**Figure6**

**Rg3 -- -- 5 10 20 μM**

**OGD/R -- + + + +**





64 KD

**keap1**





55 KD

**β-Tubulin**

**NQO1**

**β-actin**

**FTH1**

**β-Tubulin**

**GPX4**

**β-Tubulin**

**ML334 -- -- -- + +**

**OGD/R -- + + + +**

**Rg3 -- -- + -- +**



















21 KD

20 KD

33 KD

42 KD

42 KD

33 KD

55 KD

55 KD

**Raw data for figure6D and 6G in the manuscript.**

**β-actin**

**HO-1**
